# Multiple Fentanyl Overdoses — New Haven, Connecticut, June 23, 2016

**DOI:** 10.15585/mm6604a4

**Published:** 2017-02-03

**Authors:** Anthony J. Tomassoni, Kathryn F. Hawk, Karen Jubanyik, Daniel P. Nogee, Thomas Durant, Kara L. Lynch, Rushaben Patel, David Dinh, Andrew Ulrich, Gail D’Onofrio

**Affiliations:** ^1^Department of Emergency Medicine, Yale School of Medicine, New Haven, Connecticut; ^2^Department of Laboratory Medicine, Yale School of Medicine, New Haven, Connecticut; ^3^Department of Laboratory Medicine, University of California, San Francisco.

On the evening of June 23, 2016, a white powder advertised as cocaine was purchased off the streets from multiple sources and used by an unknown number of persons in New Haven, Connecticut. During a period of less than 8 hours, 12 patients were brought to the emergency department (ED) at Yale New Haven Hospital, experiencing signs and symptoms consistent with opioid overdose. The route of intoxication was not known, but presumed to be insufflation (“snorting”) in most cases. Some patients required doses of the opioid antidote naloxone exceeding 4 mg (usual initial dose = 0.1–0.2 mg intravenously), and several patients who were alert after receiving naloxone subsequently developed respiratory failure. Nine patients were admitted to the hospital, including four to the intensive care unit (ICU); three required endotracheal intubation, and one required continuous naloxone infusion. Three patients died. The white powder was determined to be fentanyl, a drug 50 times more potent than heroin, and it included trace amounts of cocaine. The episode triggered rapid notification of public health and law enforcement agencies, interviews of patients and their family members to trace and limit further use or distribution of the fentanyl, immediate naloxone resupply and augmentation for emergency medical services (EMS) crews, public health alerts, and plans to accelerate naloxone distribution to opioid users and their friends and families. Effective communication and timely, coordinated, collaborative actions of community partners reduced the harm caused by this event and prevented potential subsequent episodes.

Shortly after 4:00 p.m. on June 23, 2016, four patients with symptoms and signs of opioid overdose, characterized by central nervous system and respiratory depression, miosis (pinpoint pupil constriction), hypotension, and bradycardia, arrived in rapid succession at the York Street Campus (two patients) and St. Raphael Campus (two patients) EDs of Yale New Haven Hospital in downtown New Haven. Within 6 hours, seven additional patients arrived at the York Street Campus ED and one more at the St. Raphael ED; these patients included two who were pronounced dead on arrival and four critically ill patients requiring endotracheal intubation and ICU admission (Figure). The patients represented four geographic clusters (i.e., at least one other victim found in the same vehicle or parking lot, or in the same house or an adjacent house), and were transported by EMS crews responding to bystander 911 calls. All of the patients had clinical signs of opioid overdose and received at least one dose of naloxone from EMS (Table 1). Twelve patients met the case definition for suspected fentanyl exposure (i.e., clinical signs of opioid toxicity and response to naloxone, with laboratory confirmation of fentanyl or fentanyl metabolites in blood, or history of direct association with a laboratory-confirmed fentanyl exposure) (Table 1). Among the four patients admitted to the ICU, three required endotracheal intubation and mechanical ventilation for respiratory failure that was relatively refractory to large doses of naloxone, and one required a continuous naloxone infusion for 12 hours. Two of the three intubated patients suffered acute kidney injury and pulmonary or gastrointestinal hemorrhage, one of whom (patient K) died 3 days later from multisystem organ failure. The third patient survived with permanent cardiac injury. Other intoxicated patients who arrived at the ED with signs or symptoms of the opioid toxidrome were excluded from this analysis because of inconsistent history (e.g., patient reported using a nonfentanyl opioid) or toxicology test results that did not identify fentanyl.

**FIGURE:**
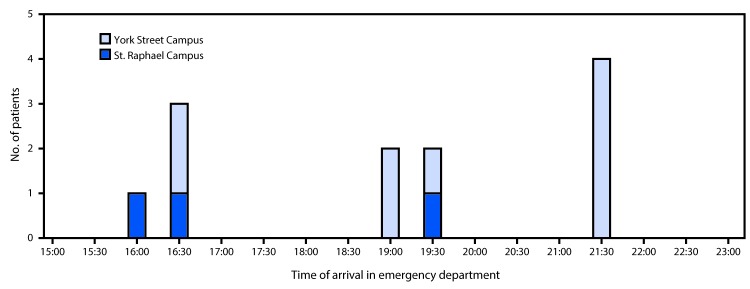
Time of arrival for 12 fentanyl overdose patients at the St. Raphael Campus (n = 3) and York Street Campus (n = 9) emergency departments of Yale New Haven Hospital — New Haven, Connecticut, June 23, 2016

**TABLE 1:** Demographic characteristics, hospital arrival time, prehospital naloxone use, and disposition for 12 patients with fentanyl overdose — Yale New Haven Hospital, New Haven, Connecticut, June 23, 2016

Patient	Age group (decade)	Sex	Arrival time	Emergency department	Naloxone (Administering provider, route)			Disposition
					(EMS, IN)	(EMS, IV/IO)	(ED/IV)	
A	60s	Male	16:16	SRC	2 mg	0	0	Discharged
B	80s	Male	16:36	YSC	2 mg	1 mg	0	Observed and discharged
C	30s	Male	16:40	YSC	3 mg	0	0.4 mg	Intensive care unit
D	40s	Male	16:48	SRC	3 mg	0	0.4 mg	Observed and discharged
E	70s	Male	19:01	YSC	4 mg	2 mg*	0	Dead on arrival in ED
F	70s	Male	19:16	YSC	2 mg	2 mg	2 mg	Observed and discharged
G	60s	Male	19:33	YSC	2 mg	2 mg	0.4 mg	Observed and discharged
H	60s	Male	19:38	SRC	2 mg	2 mg	0.4 mg^†^	Intensive care unit
I	30s	Female	21:31	YSC	0	2 mg	2 mg	Dead on arrival in ED
J	50s	Female	21:32	YSC	2 mg	1 mg	0	Intensive care unit
K	60s	Male	21:39	YSC	0	0.5 mg	0	Intensive care unit^§^
L	50s	Female	21:41	YSC	2 mg	2 mg	0	Observed and discharged

**Abbreviations:** ED = emergency department; EMS = emergency medical services; IN = intranasal; IO = intraosseous; IV = intravenous; SRC = St. Raphael Campus; YSC = York Street Campus.

* Intraosseous injection.

^†^ Naloxone drip 0.4 mg/hour for 12 hours in intensive care unit.

^§^ Patient died of multiorgan failure in intensive care unit 3 days later.

Shortly after arrival in the ED, serum toxicology screens, designed to detect a panel of nonopioid toxins, were performed for all patients, and qualitative urine immunoassay toxicology screens for drugs of abuse were performed for nine patients (A, C, D, F, G, H, J, K, and L) (Table 2). The urine immunoassay screening tests cannot detect fentanyl and its analogs; however, all but one of the nine tested positive for cocaine. The one patient with a negative urine cocaine screen (patient A) acknowledged past cocaine use. Serum and urine specimens were later analyzed at the University of California, San Francisco (UCSF) using liquid chromatography high-resolution mass spectrometry (LC-HRMS) (*1*) to detect 215 common illicit and pharmaceutical drugs and metabolites, followed by additional analyses in attempts to identify 7,038 novel drugs and metabolites (*2*,*3*). Levels of fentanyl, cocaine, benzoylecgonine (a cocaine metabolite that persists in body fluids and is an indicator of cocaine use) and levamisole (a veterinary antihelminthic that has been used as a cocaine adulterant) were quantified. Nine patients (B, C, D, F, G, H, J, K, and L) had fentanyl detected in blood that was collected during their hospitalization and tested at UCSF (Table 2). One patient who reported cocaine use before symptom onset (patient A) and who was found in the vicinity of patients B, C, and D at the time of intoxication, was discharged before the full scope of the outbreak had been recognized and did not receive confirmatory toxicology testing. The Connecticut Medical Examiner’s Office performed postmortem toxicology screens on specimens obtained from two patients who died en route to the hospital (patients E and I).

**TABLE 2:** Serum and urine toxicology test results for 12 patients with fentanyl overdose — Yale New Haven Hospital, New Haven, Connecticut, June 23, 2016

Patient	Serum levels (ng/mL)				Other substances detected	
	Fentanyl	Cocaine	BE	Levamisole	Serum	Urine
A	—*	—	—	—	—	—
B	0.9	Not detected	1	1	BE, cotinine, levamisole, norfentanyl	Specimen not available
C	0.5	0	65	13	BE, cotinine, levamisole, norfentanyl, THC-COOH	BE, cocaethylene, cocaine, cotinine, EME, levamisole, lidocaine, naloxone, nicotine, norcocaine, norfentanyl
D	0.6	0	1	1	BE, cotinine, levamisole, norfentanyl, THC-COOH	BE, cocaethylene, cocaine, EME, ethylone, hydrocodone, levamisole, naloxone, norfentanyl, THC-COOH
E^†^	11	Not detected	Not detected	—	Ethanol	—
F	4.6	2	15	2	BE, cocaethylene, cocaine, cotinine, hydroxyzine, levamisole, naloxone, norfentanyl	BE, EME, cocaethylene, cocaine, cotinine, hydroxyzine, levamisole, naloxone, norfentanyl
G	2.3	1	63	4	Acetaminophen, BE, cocaine, cotinine, levamisole, midazolam, norfentanyl, THC-COOH	α-hydroxymidazolan, acetaminophen, BE, cocaine, cotinine, EME, levamisole, midazolam, naloxone, norcocaine, norfentanyl
H	1.9	26	144	5	Acetaminophen, BE, cocaethylene, cocaine, cotinine, levamisole, naloxone, norfentanyl	Acetaminophen, BE, cocaine, cotinine, EME, hydroxyzine, levamisole, naloxone, nicotine, norcocaine, norfentanyl
I^†^	13	79	680	—	Cocaethylene, ethanol	—
J	3	26	68	6	BE, cocaine, cotinine, levamisole, naloxone, norfentanyl, tramadol	BE, cocaine, cotinine, EME, desmethyltramadol, levamisole, naloxone, norcocaine, norfentanyl, tramadol
K^§^	9.5	3	172	2	BE, cocaine, levamisole, naloxone, norfentanyl, THC-COOH	BE, cocaine, EME, levamisole, norcocaine, norfentanyl
L	3.6	4	712	64	BE, cocaethylene, cocaine, cotinine, hydroxyzine, levamisole, lidocaine, naloxone, norfentanyl	BE, cocaethylene, cocaine, cotinine, EME, hydroxyzine, levamisole, lidocaine, naloxone, norfentanyl

**Abbreviations:** BE = benzoylecgonine; EME = ecgonine methylester; THC-COOH = 11-nor-9-carboxy- tetrahydrocannabinol.

*Test not performed.

^†^ Postmortem specimens collected by medical examiner.

^§^ Died in intensive care unit.

Serum samples from the hospitalized patients analyzed at UCSF demonstrated fentanyl levels of 0.5–9.5 ng/mL (Table 2) (therapeutic range for analgesia = 0.6–3.0 ng/mL) (*4*); postmortem levels in the first two patients who died were 11 ng/mL (patient E) and 13 ng/mL (patient I). Norfentanyl, a major metabolite of fentanyl, was detected in the serum of nine patients; norfentanyl was not detected in postmortem testing of patients E and I, presumably because death occurred before metabolism of fentanyl to norfentanyl. All hospitalized patients had detectable serum levels of cocaine, cocaine metabolites (benzoylecgonine and ecgonine methyl ester), cocaethylene (a compound formed in vivo when ethanol is ingested in the presence of cocaine), or levamisole by LC-HRMS confirmatory testing (Table 2), all suggesting recent cocaine use. The absence of other opioids, such as heroin, methadone, or oxycodone, in serum (only one patient [D] was hydrocodone positive) was consistent with reports by the patients that most were not habitual opioid users.

Additional substances detected in serum and urine were reported qualitatively (Table 2) and reflected nicotine (cotinine), cannabinoid (tetrahydrocannabinol), and hydroxyzine (antihistamine) use, or receipt of naloxone. Postmortem toxicology screens identified fentanyl as a cause of death for patients E and I, both of whom arrived in the ED in cardiac arrest. In addition to the clinical specimens, one 32-mg forensic sample of the illicit drug material collected by law enforcement was tested at the Drug Enforcement Administration laboratory. Analysis of that product recovered from an involved crime scene found 6.6% (± 0.8%) fentanyl by weight with trace amounts of cocaine and an inert adulterant.

Within a few hours of recognition of the outbreak, a multiagency response involving the New Haven Office of Emergency Management, New Haven and Connecticut Departments of Public Health, the Drug Enforcement Administration, local police, Connecticut Poison Control Center, and the New Haven Mayor’s Office was undertaken. Initial actions included 1) rapid notification of public health and law enforcement agencies by ED and EMS personnel; 2) real-time interviews of patients and family members in an attempt to trace and limit further use or distribution of the fentanyl; 3) advice to EMS crews to increase naloxone doses in treating suspected cases; 4) public health alerts regarding the event, including notices of the sale of a high potency opioid marketed as cocaine causing deaths in the region; and 5) plans to accelerate distribution of naloxone to opioid users and their friends and families. The high naloxone requirements necessitated both immediate naloxone resupply and augmentation for local EMS crews, including the transfer of 700 naloxone kits from the Connecticut Department of Public Health to hospitals and EMS crews the following morning. Actions of multiple partners led to the arrest 4 days later of three persons allegedly responsible for the illicit fentanyl sales.

## Discussion

This explosive occurrence of multiple fentanyl overdoses triggered a rapid response by public safety and medical communities to identify the substance and its source. Federal, state, and local agencies responded to confine the outbreak quickly, save patient lives where possible, alert the public, and gather additional information. The rapid medical, law enforcement, and public health actions likely limited the extent and impact of this outbreak.

These events highlight the intrinsic risks inherent in illicit drug use and support the broad distribution of naloxone. The urine toxicology screens suggest that most patients were cocaine users, but not chronic opioid users, and as such, would likely not have received any training in the identification or treatment of opioid overdose. This episode resulted in the formation of a partnership between the Connecticut Department of Public Health and Yale New Haven Hospital that facilitated implementation of a pilot program to provide overdose education and take-home naloxone kits to ED patients at risk for overdose. In addition, community opioid treatment programs and providers collaborated with the EDs to provide rapid access to treatment for patients with opioid use disorders.

Commonly available immunoassay toxicology screening tests are unable to detect fentanyl or its metabolites; the opiate screen is designed to detect codeine, morphine, and heroin, and with an expanded panel, oxycodone and methadone. Widespread use of toxicology screens unable to detect fentanyl or its analogs underscores the importance of recognizing the opioid toxidrome. Rescuers and clinicians should recognize the potential need to administer multiple or high doses of naloxone in cases of opioid overdose that do not respond to administration of a single standard naloxone dose where fentanyl or its analogs (highly potent opioids) might be responsible for unresponsiveness. The total dose of naloxone required for opioid reversal will depend on many factors, including the opioid dose, the potency of the opioid in binding receptors, the lipophilicity of the opioid in crossing into the central nervous system, the elimination half-life of the opioid, individual patient factors, and the route of administration of the naloxone (intranasal compared with intramuscular or intravenous) (*5–7*). Because of the persistent respiratory depression associated with fentanyl, additional doses of naloxone might be needed after initial reversal.*

Although illicit opioids often are mixed with harmful adulterants (e.g., fentanyl and its analogs blended with or deliberately substituted for heroin or mixed with the opioid analgesic combination of acetaminophen and hydrocodone [e.g., Norco]) (*8**,**9*), this outbreak was unique in representation of fentanyl as cocaine to an opioid-naïve population, which resulted in an outbreak of fatal and nonfatal overdoses.

Lack of metabolism of fentanyl to norfentanyl might be the result of rapid death after fentanyl use (*10*). It has been suggested that rapid death might be caused by immediate onset of respiratory arrest or that fentanyl might cause rapid onset of chest wall rigidity, leading to death (*10*). This effect of fentanyl is well recognized by clinicians familiar with the drug, but is not likely to be known among illicit drug users. In addition, many users might be unaware that their expected substance of choice might be substituted by or adulterated with high doses of fentanyl.

Distribution of naloxone to persons at risk for opioid overdose, their families, and friends through prescriptions by practitioners, pharmacists, and other public health avenues might help prevent fatal fentanyl overdoses. In addition, this outbreak of severe opioid intoxication among patients who were cocaine users, but not chronic opioid users, suggests that distributing naloxone and offering training to all illicit drug users, their friends, and family members might prevent such opioid-associated morbidity and mortality. The swift coordinated multiagency response likely limited the impact of this outbreak, and the resultant strengthening of community partnerships has the potential to further limit the morbidity and mortality related to opioids in communities.

**Summary**

**What is already known about this topic?**

Fentanyl and its analogs have been substituted for heroin and other opioids, and are usually marketed to persons seeking opioids. Because of fentanyl’s high potency compared with heroin, methadone, and oxycodone, there is a high risk for fatal overdose associated with illicit use. Higher than normal doses of the opioid antagonist naloxone might be required to reverse fentanyl overdose.

**What is added by this report?**

On June 23, 2016, fentanyl marketed as cocaine resulted in an extraordinary opioid overdose outbreak in New Haven, Connecticut, resulting within 6 hours in at least 12 cases, marked by four intensive care unit admissions and three deaths. A rapid and coordinated public health response involving multiple partners likely reduced the impact of this outbreak.

**What are the implications for public health practice?**

A collaborative and timely multi-organization response can mitigate the consequences of an extraordinary public health event. Development and implementation of a screening test for fentanyl might inform clinicians about the presence of these particularly deadly opioids and prevent deaths. Opioid use education and naloxone administration kits and education should be extended to all persons at risk for illicit drug use, their families, and friends.
